# Personality traits and health-related behaviors in medical students facing a stressful event

**DOI:** 10.3389/fpubh.2023.1256883

**Published:** 2023-10-10

**Authors:** Julita Tokarek, Angelika Kapuścik, Joanna Kućmierz, Edward Kowalczyk, Michał Seweryn Karbownik

**Affiliations:** ^1^Department of Pharmacology and Toxicology, Students Research Club, Medical University of Lodz, Lodz, Poland; ^2^Students Research Club “Metoda”, Institute of Sociology, Faculty of Economics and Sociology, University of Lodz, Lodz, Poland; ^3^Department of Pharmacology and Toxicology, Medical University of Lodz, Lodz, Poland

**Keywords:** personality traits, conscientiousness, diet, health, behaviors, psychological stress

## Abstract

**Background:**

It is believed that personality traits have an impact on the propensity to change and maintain favorable lifestyle habits. This issue has been raised by multiple studies, however, none of them appeared to focus on population under severe psychological stress. The aim of the present study was to investigate the link between personality traits and health-related behaviors and measures such as dietary intake of specific food products, physical activity, body-mass index and the use of cigarettes in medical students facing a stressful event.

**Methods:**

The study included a cohort of third-year medical students from the Medical University of Lodz, Poland, facing a stressful subject exam during the first COVID-19-related lockdown. At baseline, personality traits were evaluated with the use of the Polish version of the Big Five Inventory-Short questionnaire. Then, consumption of selected food products was monitored with the use of seven-day electronic dietary record. Also, some other health-related data was collected (body-mass index, physical activity and the use of cigarettes). General Linear Modeling techniques, logistic regression and exploratory factor analysis were applied to analyze the data.

**Results:**

Four hundred and forty-four students completed the study. A two-factor pattern of food consumption was discovered by the exploratory factor analysis in the study group (34% of the variance explained). Higher conscientiousness, but not the other personality traits, was found to be significantly associated with generally healthier lifestyle manifested by higher consumption of vegetables, wholegrain products, fruits and nuts (adjusted beta 0.16, 95%CI 0.06 to 0.26, pη^2^ = 2.3%, *p* = 0.0015) and lower cigarette smoking (adjusted odds ratio 0.84, 95%CI 0.75 to 0.94, *p* = 0.0020), but insignificantly with physical activity and body-mass index.

**Conclusion:**

Severely stressed medical students expressing high conscientiousness tend to present healthier behaviors. Therefore, interventions aimed at improving lifestyle habits in students with low conscientiousness might be useful.

## 1. Introduction

The average lifespan has increased in the past few decades due to socioeconomic and health-related development. As a result, it has led to a higher prevalence of chronic old-age diseases such as diabetes, obesity, cardiovascular diseases and cancer. Many of these diseases are conditioned by the modifiable risk factors including smoking, physical activity, alcohol intake, body weight and diet (1). Thereupon, changing and maintaining favorable lifestyle behaviors as early as possible in people’s lives are a key strategy for good health and longevity.

Leading a healthy lifestyle may be challenging under psychological stress, which is very prevalent contemporarily. Under psychological stress, attention is redirected to stressor-related issues and dietary behaviors may be neglected (2). In particular, undergraduate students face multiple psychological stressors that may negatively affect their health behaviors (3). Recently, the general stress associated with academic workload has been further exacerbated by the COVID-19 pandemic and global lockdown (4); distancing from family members and friends, thereby diminished social interaction and emotional support together with new online teaching and assessment methods, which required appropriate digital infrastructure and skillset, have led to unprecedented psychosocial burden (5, 6). The emergent problems appeared to heterogeneously affect health-related behaviors (7), which, in turn, could depend on some personal factors (8).

Personality refers to individual differences in characteristic patterns of thinking and is one of the most significant aspects of human life. It has an influence on all human behaviors in both personal and social life (9). Personality development is a complex process influenced by factors such as genetics, environmental factors and temperament which is a precursor of the structure of personality (10). Personality traits may have an impact on propensity to change and maintain favorable lifestyle habits. This issue has been raised by multiple studies to highlight the link between personality traits and the tendency to consume specific food groups (11). In particular, neuroticism was associated with unhealthy diet habits such as low consumption of fruits and vegetables, and higher consumption of sugar and saturated fats (11). On the other hand, conscientiousness was found to be linked with higher scores on the health aware diet factor and higher intake of fruits and vegetables (11–13). Even though similar studies have already been conducted, none of them focused specifically on a population under severe psychological stress. In fact, the recent meta-analysis on this topic showed that further research is required to explore this relationship in more vulnerable groups (11).

The primary aim of the present study was to investigate the link between personality traits and dietary intake of specific food products in medical students facing a stressful subject examination in the first COVID-19-related lockdown. In addition, the other health-related measures (general quality of diet, physical activity, body-mass index and cigarette use) were also assessed as secondary outcomes in terms of their relationship to personality traits.

## 2. Methods

### 2.1. Ethical considerations

The study obtained the approval of the Bioethics Committee of the Medical University of Lodz, Poland (RNN/111/20/KE, received on April 2, 2020). All the participants gave their informed consent in an electronic manner. The study partially represents a secondary analysis of already published results (14, 15), however, it also includes new data.

### 2.2. Research design

In this study medical students were exposed to the growing stress related to an impending final subject examination. The study was performed during the first COVID-19 pandemic wave in Poland. At that time, several unprecedented social restriction measures had lasted continuously for 2–3 months in the country: suspension of stationary educational activities, travel restrictions and gathering ban, closure of non-essential retail outlets and services, ban on traveling alone for juveniles and obligation to wear masks (16). Although the number of COVID-19 cases did not exceed 500 a day at that time in Poland, the imposed lockdown measures and the surrounding atmosphere of insecurity could have deteriorated mental functioning of the study’s participants even more.

**Figure 1:**
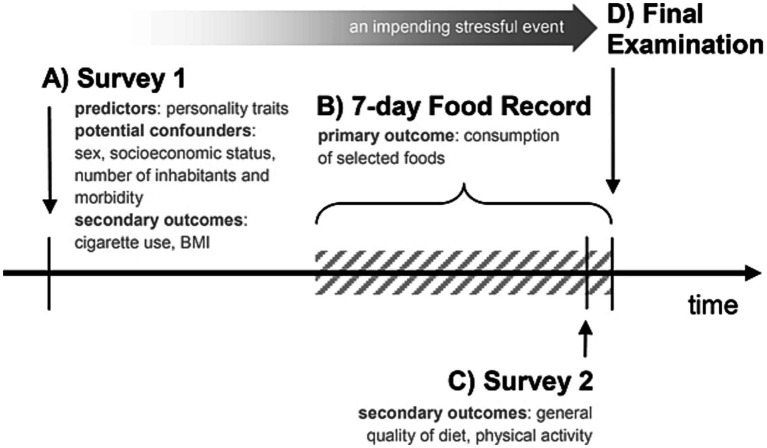
Timeline of the study procedures. Four steps of the study are indicated with letters A–D. All the participants were exposed to the growing stress accompanying the im-pending final exam (D). At the entrance, personality traits of the participants were assessed as predictors (A). The primary outcome measure of consumption of selected foods had been recorded for 7 days (B) and, in the last day, physical activity and general quality of diet were assessed as secondary outcomes (C). Some secondary outcome measures were evaluated also in a cross-sectional manner, at the entrance (A): cigarette smoking/use and the body-mass index (BMI). Some potential confounders (A) were also assessed.

This was a survey-based study. Personality traits were evaluated as predictors in the entrance survey, whereas consumption of selected foods was recorded as the primary outcome measure during 7 days preceding the final exam. Physical activity and general quality of diet were assessed as secondary outcomes a day before the final exam. Some secondary outcome measures (cigarettes, body-mass index) were evaluated in a cross-sectional manner at the entrance to the study (Figure 1).

The timeline of the study was as follows (Figure 1). After (A) collecting baseline sociodemographic and anthropometric characteristics, some health-related habits and personality traits as predictors (4–7 June 2020), the students were (B) followed up for 7 days in terms of their dietary behaviors (9–15 June 2020). Also, (C) pre-exam physical activity and general quality of diet were assessed in the last day of dietary recording (14–15 June 2020) – this period preceded (D) a stressful final subject exam (15 June 2020). Participants were recruited to the study for 2 weeks prior to its initiation.

The STROBE (STrengthening the Reporting of OBservational studies in Epidemiology) guidelines for reporting cohort studies (17) were applied to outline the study description.

### 2.3. Participants

The study included a cohort of third-year medical students of the Medical University of Lodz, Poland, taking the 60-min pharmacology exam in June 2020. No exclusion criteria were applied in the study. Students were approached during their pharmacology lecture and were invited to join the pre-study online meeting (Microsoft Teams; Microsoft, Redmont, WA, USA), when the general concept of the study was explained. MS Teams platform was also used to facilitate communication throughout the study and to distribute the surveys.

### 2.4. Instruments

The listed Instruments were used in the course of the study:

- to assess predictors – personality traits:

o Polish version of the Big Five Inventory-Short (BFI-S) questionnaire (18, 19);

BFI-S assesses five dimensions of personality with the use of three items to each; participants are asked to express the extent to which they agree with each of the items in a seven-point Likert scale. Cronbach’s alpha assessed in the study sample for the subscales was 0.65 for Neuroticism, 0.75 for Extraversion, 0.70 for Openness, 0.54 for Agreeableness and 0.68 for Conscientiousness.

- to assess primary outcome – food consumption:

o Food Record questionnaire

This was a seven-day selective dietary record questionnaire using the open-ended electronic Food Record (FR) form. Although the tool was previously used (14, 15), in the current study we performed its further validation, which confirmed its satisfactory accuracy, but somehow variable precision. Details of the methods used in the validation process and its results are reported in Supplementary material S1.

The FR was designed to collect the information regarding the mass of 34 food categories:

1. Meat, sausages, fish, seafood

- red meat
- white meat
- fatty fish
- other fish
- fish oil
- seafood

2. Dairy and eggs

- milk
- cottage cheese
- cheese
- yogurt, kefir, soured milk
- eggs

3. Grain products

- light bread
- wholemeal bread, graham
- cereal, groats, whole grain noodle
- muesli
- white rice
- unpasteurized kvass and beer
- wholemeal flour

4. Vegetables

- potatoes
- carrot, parsley, celery
- beetroots
- raw cucumber
- pickled cucumber and pickling juice
- cabbage
- sauerkraut and pickling juice
- other fermented vegetables and their pickling juice
- onion, leek, garlic
- leguminous vegetables
- all other vegetables

5. Fruits and nuts

- apples
- citrus
- bananas
- all other fruits
- nuts

Each of the food categories in the FR was described in detail and illustrated with several photos of food portion examples to enable mass estimation. The participants were asked to self-report the food quantity by filling the online FR form each time they consumed a meal/beverage within 7 days. In this study, consumption of the following foods was assessed as a sum of the abovementioned categories (indicated below in the brackets) and was used as the outcome measure:

- red meat (1a),
- white meat (1b),
- fish, fish oil and seafood (marine products) (1c – 1f),
- dairy (2a – 2d),
- eggs (2e),
- wholegrain products (3b, 3c, 3 g),
- light bread (3a),
- potatoes (4a),
- vegetables (4b – 4 k),
- fruits (5a – 5d),
- nuts (5e);

- to assess secondary outcomes:

o anthropometric questionnaire (body mass and height to calculate body-mass index);
o self-reported cigarette smoking or use of e-cigarettes;
o Polish version of the Starting the Conversation (STC) scale to assess general quality of diet (20), STC is composed of eight questions relating to consumption of selected foods and beverages; participants indicate frequency of consumption over several previous months using a three-point Likert scale; this was used for the purpose of comparison with the pre-exam quality of diet;
o single-item five-point semantic differential scale from 1 (“I have no physical activity at all”) to 5 (“I play sports intensively five times a week”) to assess physical activity, partially validated (16) against the Polish version of the International Physical Activity Questionnaire Short form (21, 22);

- to assess potential confounders:

o sociodemographic data questionnaire (year of birth, sex, socioeconomic status – measured subjectively using a single-item three-point Likert scale: low, middle, high – number of inhabitants in the place of family residence);
o self-reported morbidity (suffering from chronic diseases);

- to assess symptoms of depression:

o Polish version of the Patient Health Questionnaire-9 (PHQ-9) scale (23, 24);

- to assess symptoms of anxiety:

o Polish version of the Generalized Anxiety Disorder-7 (GAD-7) scale (25, 26);

PHQ-9 and GAD-7 assess the symptoms based on nine- and seven-symptom lists, respectively; participants indicate frequency of experiencing each of the symptoms with a four-point Likert scale; original tools apply a two-week time frame, however, for the purpose of this study, a one-week time frame of experiencing the symptoms was used (27, 28) to capture the critical time of examined health-related behavior (seven-day food record).

### 2.5. Procedure

#### 2.5.1. Step A

Firstly, basal data on the participants were collected using the Internet survey (Step A in Figure 1 – Survey 1; Google Form; Google, Mountain View, CA, USA). The survey included among others:

- general information: sociodemographic, anthropometric and morbidity (suffering from chronic diseases); the data was used for descriptive purpose as well as to get potential confounders (sex, socioeconomic status, number of inhabitants and morbidity), effect modifiers (morbidity) and cross-sectional outcome measure (BMI);
- health-related habits:

o baseline physical activity, used for the purpose of comparison with the pre-exam physical activity;
o baseline general quality of diet;
o cigarette smoking or use of e-cigarettes, a cross-sectional outcome measure;

- personality traits, used as the predictor variable in the analysis.

#### 2.5.2. Step B

Secondly, a few days after the baseline survey was conducted, the seven-day selective dietary record questionnaire using the open-ended electronic Food Record form (FR; Step B in Figure 1; Google Form) was applied.

#### 2.5.3. Step C and D

Thirdly, mental health symptoms (depression and anxiety) were evaluated in the last day of food recording (a day before the final subject exam) to confirm the severity of mental burden related to the impending stressful event (Step C in Figure 1). The stressor (final exam in pharmacology – step D in Figure 1) was previously demonstrated to elicit physiological (heart rate) and psychological (state anxiety) stress response and its hormonal effect (rise in salivary cortisol) was notable as early as a day before (29). Mental health was also found to slightly deteriorate a few days before the exam (14). In addition, in the survey performed a day before the exam (Step C), pre-exam physical activity and pre-exam general quality of diet (STC) were evaluated as outcome measures using the same tool as in the first study step (adjusted to one-week time).

### 2.6. Data analysis

Baseline characteristics of the study participants were described by mean with standard deviation, median with 1^st^-3^rd^ quartiles or absolute number with frequency for continuous, ordinal and categorical variables, respectively. General Linear Modeling (GLM) techniques were applied to assess the association between personality traits and health-related behavior (consumption of specific foods and physical activity). Cigarette use was assessed with logistic regression. Apart from the raw multivariate regression results, the multivariate regression analyses adjusted for potential confounders (sex, socio-economic status, number of inhabitants in the place of family residence and any chronic disease) were reported – these covariates were selected as potentially affecting both the predictors and outcomes; potential confounders were included as independent variables linearly linked to the outcome in the statistical models. A separate multivariate linear regression model was used for every food product. The Benjamini and Hochberg (B-H) correction for multiple comparisons was applied with the False Discovery Rate of 0.05. To identify potential links between health-related behaviors, exploratory factor analysis (EFA) with raw varimax factor rotations as well as hierarchical agglomerative clustering with Euclidean distance and complete-linkage were used. *p*-values below the B-H corrected significance levels were considered statistically significant. The analyses were performed using STATISTICA Software version 13.3 (Statsoft; Tulsa, OK, USA).

Due to the fact that no forced answering option was used in all the survey items, some missing data occurred in the database. The missingness pattern was assumed to be random. Missing data comprised 67/34,596 (0.19%) of the values in the database. All the missing values were imputed before any analysis with the use of Multiple Imputation by Chained Equations (MICE) method.

## 3. Results

### 3.1. Characteristics of the study sample

Four hundred and ninety students volunteered for the study. Forty-six participants (9.4%) dropped out during the course of the study (not providing food records for at least 3 days or not filling Survey 2). The number of the participants who completed the study was 444 and only data on these subjects was evaluated. The mean age of the participants was 22.7 and one third of them was male. Detailed characteristics of the study sample are presented in Table 1.

**Table 1:** Basal characteristics of the study participants (*n* = 444).

Characteristics	Mean (standard deviation), median (1st–3rd quartiles) or absolute number (frequency)
**Sociodemographic and physical data**	
Age	
[years]	22.7 (1.1), median (Q1–Q3): 22.0 (22.0–23.0)
Sex	
Female	297 (66.9%)
Male	147 (33.1%)
Socioeconomic status	
Low	3 (0.7%)
Middle	313 (70.5%)
High	128 (28.8%)
Number of inhabitants in a place of family residence	
Below 5,000	103 (23.2%)
5,000–50,000	139 (31.3%)
50,000–500,000	113 (25.5%)
Over 500,000	89 (20.0%)
Anthropometry	
Body mass index [kg × m^−2^]	22.0 (3.1), median (Q1-Q3): 21.5 (19.6–23.6)
Health-related behavior	
Current cigarette smoking/use^a^	29 (6.5%)
**Morbidity**	
Any chronic disease	172 (38.7%)
Allergic diseases	106 (23.9%)
Neurological diseases	6 (1.4%)
Psychiatric diseases	26 (5.9%)
Cardiological diseases	5 (1.1%)
Gastroenterological diseases	28 (6.3%)
Immunological diseases	8 (1.8%)
Cancerous diseases	1 (0.2%)
Endocrine diseases	42 (9.5%)
**Personality traits** ^**b**^	
Neuroticism	13 (9 – 16)
Extraversion	12 (9 – 15)
Openness	15 (12 – 17)
Agreeableness	15 (12 – 17)
Conscientiousness	16 (13 – 18)

Q1, 1st quartile. Q3, 3rd quartile. ^a^Fraction of the participants reporting either traditional cigarette smoking or e-cigarette use. ^b^Expression of five personality traits was presented numerically in the range of 3–21, with the midpoint of 12.

### 3.2. Pre-exam mental health symptoms

As assumed, in the assessed pre-exam time period worrisome mental symptoms were observed: 35% of the students were characterized by mild depressive symptoms (PHQ-9 of 5–9) and additional 40% by moderate or more severe depressive symptoms (PHQ-9 ≥ 10). Furthermore, 35% of the students presented mild and another 34% moderate or more severe anxiety symptoms (GAD-7 of 5–9 and ≥10, respectively). Median (1st–3rd quartile) for depressive symptoms was 8 (4.5–12) and for anxiety symptoms 7 (4–12.5).

### 3.3. Pre-exam health-related behavior

Physical activity and general quality of diet were assessed twice in the study: at the entrance in relation to several months and a day before the stressful exam in relation to a previous week. The participants reported being significantly less physically active in the seven-day pre-exam period than at baseline (Wilcoxon signed-rank test, *p* < 0.0001), however, they presented relatively similar dietary practices to those at baseline as assessed by the STC score comparisons (Wilcoxon signed-rank test, *p* = 0.13).

As compared to other food groups, vegetables were the category of food products consumed in the highest amount; however, it could be even higher to comply with the common dietary guidelines (30). The students consumed a sufficient amount of fruits and dairy, as recommended (30). The fact that the study was conducted in June might have had an impact on these results because of many seasonal fruits and vegetables available. Furthermore, the consumption of marine products and nuts was insufficient to cover the demand for omega-3 fatty acids (30). Detailed data are presented in Table 2, whereas data on consumption of all food products included in the Food Record questionnaire in the seven-day pre-exam period are presented in Supplementary material – point 2.

**Table 2:** Consumption of the analyzed food products in the seven-day pre-exam period.

FR item number	Food product	Seven-day consumption [gram]	
		Median (1st–3rd quartiles)	Mean (standard deviation)
1a	Red meat	380.8 (134.4–686.2)	474.2 (445.1)
1b	White meat	336.0 (75.6–697.2)	460.5 (464.4)
1c – 1f	Marine products	65.1 (0.0–224.0)	145.5 (197.7)
2a – 2d	Dairy	1327.2 (784.0–2005.4)	1545.6 (1110.2)
2e	Eggs	246.4 (123.2–409.4)	288.3 (238.0)
3b, 3c, 3 g	Wholegrain products	641.2 (314.3–972.7)	730.1 (585.7)
3a	Light bread	514.3 (230.7–842.9)	569.2 (425.4)
4a	Potatoes	426.9 (201.6–672.0)	469.7 (369.0)
4b – 4 k	Vegetables	1637.1 (1044.4–2438.8)	1847.2 (1110.6)
5a – 5d	Fruits	1270.0 (634.4–1975.6)	1468.0 (1062.8)
5e	Nuts	15.7 (0.0–67.2)	50.9 (81.6)

### 3.4. Association between personality traits and health-related behavior under stress

In both the raw analysis and the analysis adjusted for covariates (sex, socioeconomic status, number of inhabitants in the place of family residence and any chronic disease), consumption of wholegrain products as well as the general quality of diet in the stressful period was found to be significantly positively associated with higher conscientiousness. Also, cigarette smoking appeared to be significantly negatively associated with this personality trait. Consumption of other food groups as well as physical activity were found insignificantly linked to personality traits. Detailed results of the covariate-adjusted associations are presented in Table 3, whereas the raw results in Supplementary material – point 3. Moreover, several sociodemographic covariates (used in the adjusted models) were linked to eating behaviors as detailed in Supplementary material – point 4.

**Table 3:** Covariate-adjusted association between personality traits and health-related behavior under stress.

Health-related behavior	Effect size measures and *p*-value				
	Neuroticism	Extraversion	Openness	Agreeableness	Conscientiousness
**Consumption of selected food products – primary outcome**					
Red meat	0.00 (−0.09 to 0.09)	0.01 (−0.08 to 0.09)	−0.02 (−0.10 to 0.07)	0.01 (−0.08 to 0.09)	0.02 (−0.07 to 0.10)
	pη^2^ = 0.0% *p* = 0.96	pη^2^ = 0.0% *p* = 0.84	pη^2^ = 0.0% *p* = 0.68	pη^2^ = 0.0% *p* = 0.89	pη^2^ = 0.0% *p* = 0.71
White meat	0.12 (0.02 to 0.22)	−0.00 (−0.09 to 0.09)	−0.10 (−0.19 to −0.00)	0.00 (−0.09 to 0.10)	0.08 (−0.01 to 0.18)
	pη^2^ = 1.3% *p* = 0.016	pη^2^ = 0.0% *p* = 0.99	pη^2^ = 0.9% *p* = 0.048	pη^2^ = 0.0% *p* = 0.95	pη^2^ = 0.6% *p* = 0.10
Marine products	−0.07 (−0.17 to 0.03)	0.13 (0.03 to 0.22)	0.05 (−0.04 to 0.15)	−0.07 (−0.17 to 0.03)	0.11 (0.01 to 0.21)
	pη^2^ = 0.4% *p* = 0.19	pη^2^ = 1.5% *p* = 0.010	pη^2^ = 0.3% *p* = 0.27	pη^2^ = 0.5% *p* = 0.15	pη^2^ = 1.1% *p* = 0.029
Dairy	−0.01 (−0.11 to 0.10)	0.02 (−0.08 to 0.12)	0.02 (−0.08 to 0.12)	−0.03 (−0.13 to 0.07)	0.12 (0.02 to 0.22)
	pη^2^ = 0.0% *p* = 0.93	pη^2^ = 0.0% *p* = 0.67	pη^2^ = 0.0% *p* = 0.67	pη^2^ = 0.1% *p* = 0.53	pη^2^ = 1.2% *p* = 0.020
Eggs	0.06 (−0.04 to 0.16)	−0.04 (−0.14 to 0.05)	−0.02 (−0.12 to 0.07)	0.05 (−0.05 to 0.15)	0.11 (0.01 to 0.20)
	pη^2^ = 0.4% *p* = 0.88	pη^2^ = 0.2% *p* = 0.21	pη^2^ = 0.1% *p* = 0.37	pη^2^ = 0.2% *p* = 0.30	pη^2^ = 1.0% *p* = 0.036
Wholegrain products	−0.02 (−0.12 to 0.08)	0.02 (−0.08 to 0.12)	0.03 (−0.06 to 0.13)	−0.02 (−0.12 to 0.08)	**0.15 (0.05 to 0.25)**
	pη^2^ = 0.0% *p* = 0.72	pη^2^ = 0.0% *p* = 0.69	pη^2^ = 0.1% *p* = 0.50	pη^2^ = 0.0% *p* = 0.65	**pη**^**2**^ **= 2.0% *p* = 0.0029**
Light bread	−0.00 (−0.10 to 0.09)	−0.06 (−0.15 to 0.03)	−0.04 (−0.14 to 0.05)	−0.02 (−0.11 to 0.08)	−0.07 (−0.16 to 0.02)
	pη^2^ = 0.0% *p* = 0.94	pη^2^ = 0.4% *p* = 0.20	pη^2^ = 0.2% *p* = 0.38	pη^2^ = 0.0% *p* = 0.70	pη^2^ = 0.5% *p* = 0.14
Potatoes	0.07 (−0.03 to 0.17)	−0.01 (−0.11 to 0.08)	0.03 (−0.07 to 0.13)	−0.04 (−0.14 to 0.05)	0.03 (−0.06 to 0.13)
	pη^2^ = 0.5% *p* = 0.15	pη^2^ = 0.0% *p* = 0.80	pη^2^ = 0.1% *p* = 0.53	pη^2^ = 0.2% *p* = 0.39	pη^2^ = 0.1% *p* = 0.51
Vegetables	−0.09 (−0.19 to 0.01)	0.03 (−0.06 to 0.13)	0.07 (−0.03 to 0.17)	0.06 (−0.04 to 0.15)	0.06 (−0.04 to 0.16)
	pη^2^ = 0.7% *p* = 0.080	pη^2^ = 0.1% *p* = 0.49	pη^2^ = 0.5% *p* = 0.15	pη^2^ = 0.3% *p* = 0.25	pη^2^ = 0.3% *p* = 0.24
Fruits	−0.10 (−0.20 to 0.00)	0.02 (−0.08 to 0.12)	−0.06 (−0.15 to 0.04)	−0.02 (−0.11 to 0.08)	0.08 (−0.01 to 0.18)
	pη^2^ = 0.8% *p* = 0.056	pη^2^ = 0.0% *p* = 0.70	pη^2^ = 0.3% *p* = 0.27	pη^2^ = 0.0% *p* = 0.75	pη^2^ = 0.6% *p* = 0.095
Nuts	−0.07 (−0.17 to 0.03)	−0.03 (−0.13 to 0.07)	0.01 (−0.08 to 0.11)	−0.00 (−0.10 to 0.09)	0.01 (−0.09 to 0.11)
	pη^2^ = 0.4% *p* = 0.18	pη^2^ = 0.1% *p* = 0.53	pη^2^ = 0.0% *p* = 0.77	pη^2^ = 0.0% *p* = 0.93	pη^2^ = 0.0% *p* = 0.79
**Other health-related measures – secondary outcomes**					
Physical activity^**a**^	−0.10 (−0.20 to 0.00)	−0.01 (−0.10 to 0.09)	0.09 (−0.01 to 0.19)	−0.01 (−0.11 to 0.08)	0.10 (−0.04 to 0.15)
	pη^2^ = 0.8% *p* = 0.056	pη^2^ = 0.0% *p* = 0.91	pη^2^ = 0.7% *p* = 0.071	pη^2^ = 0.0% *p* = 0.78	pη^2^ = 0.3% *p* = 0.027
General quality of diet^**b**^	0.01 (−0.11 to 0.09)	0.07 (−0.16 to 0.03)	0.01 (−0.11 to 0.09)	−0.03 (−0.07 to 0.13)	**0.19 (−0.29 to − 0.09)**
	pη^2^ = 0.0% *p* = 0.82	pη^2^ = 0.4% *p* = 0.18	pη^2^ = 0.0% *p* = 0.88	pη^2^ = 0.1% *p* = 0.52	**pη**^**2**^ **= 3.3% *p* = 0.0001**
Cigarettes^**c**^	0.99 (0.89 to 1.1)	1.05 (0.96 to 1.17)	0.94 (0.84 to 1.04)	0.89 (0.79 to 1.01)	**0.84 (0.75 to 0.94)**
	*p* = 0.91	*p* = 0.26	*p* = 0.22	*p* = 0.064	***p* = 0.0020**
BMI	−0.11 (−0.21 to −0.02)	−0.04 (−0.13 to 0.05)	−0.04 (−0.13 to 0.05)	0.00 (−0.09 to 0.09)	−0.08 (−0.17 to 0.01)
	pη^2^ = 1.3% *p* = 0.017	pη^2^ = 0.2% *p* = 0.38	pη^2^ = 0.2% *p* = 0.40	pη^2^ = 0.0% *p* = 0.99	pη^2^ = 0.7% *p* = 0.09

Consumption of selected food products, physical activity, general quality of diet and BMI were assessed with the use of multivariate linear regression and standardized ß with 95% confidence intervals (CI) and partial eta-square were presented as effect size measures. Cigarettes use was assessed with the use of multivariate logistic regression and odds ratio with 95% CI were presented as effect size measures. Each raw in the table depicts the results of a single multivariate model adjusted for sex, socioeconomic status, number of inhabitants in the place of family residence and the fact of any chronic disease. BMI – body-mass index. The values in bold are considered statistically significant at the Benjamini and Hochberg corrected significance level of 0.0037 (false discovery rate 0.05).^a^Physical activity was assessed in the pre-exam stage of the study and is expressed in the five-point semantic differential scale from 1 (“I have no physical activity at all”) to 5 (“I play sport intensively 5 times a week”), with the midpoint of 3.^b^General quality of diet was assessed in the pre-exam stage of the study and is expressed as the number of points in the reverse-coded Starting the Conversation scale in the range from 0 (maximally unhealthy diet) to 16 (maximally healthy diet), with the midpoint of 8; Cronbach’s alpha assessed in the study sample was 0.55.^c^Odds ratio with 95% confidence intervals for reporting either traditional cigarette smoking or e-cigarette use.

**Figure 2:**
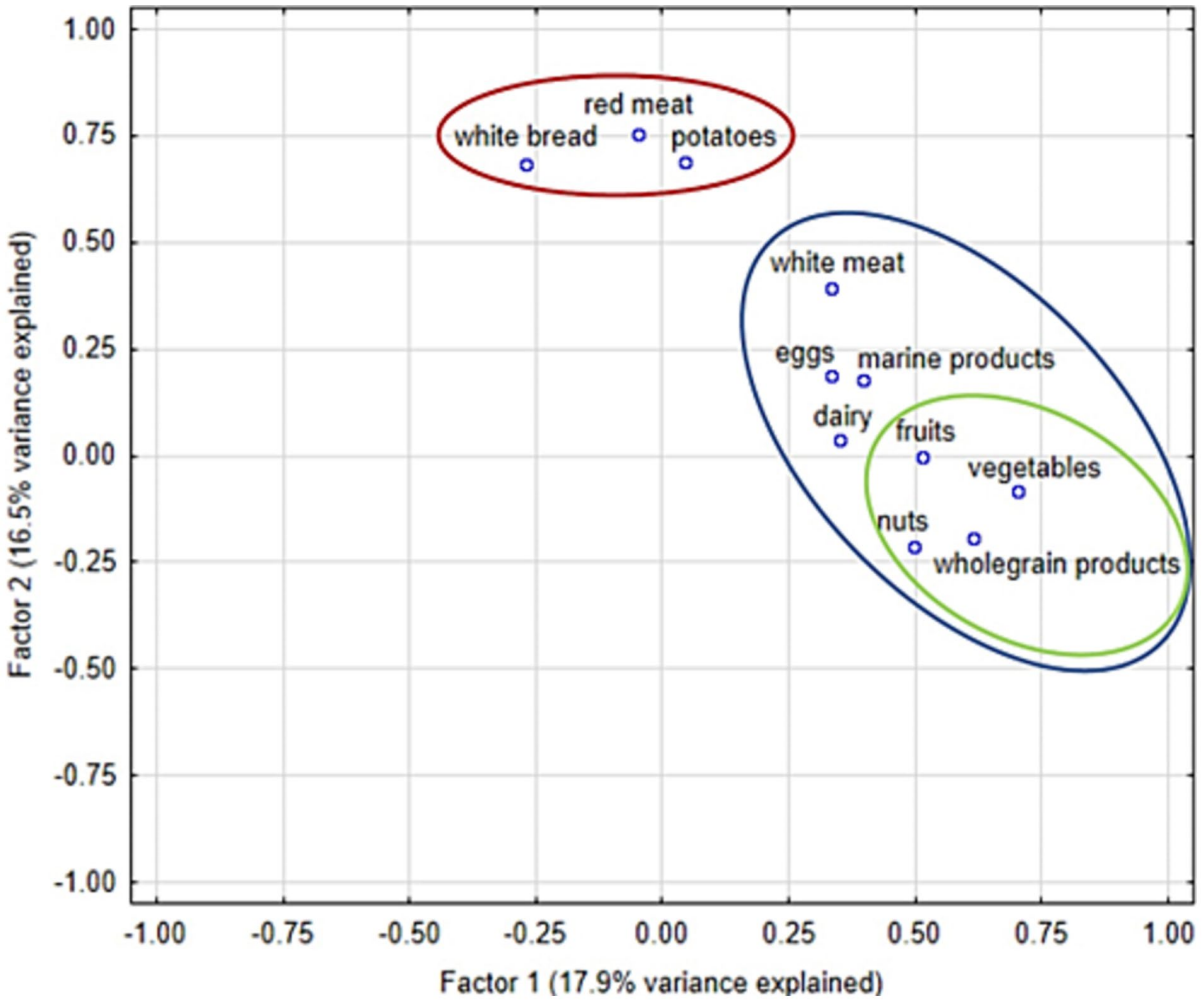
The results of the exploratory factor analysis (EFA) between 11 consumed food categories. Factor 1 – EFA-derived representation of products with higher nutritional value (blue ellipse – products included in Factor 1; green ellipse – products with highest loadings within Factor 1). Factor 2 – EFA-derived representation of products with lower nutritional value (red ellipse – products included in Factor 2).

**Figure 3:**
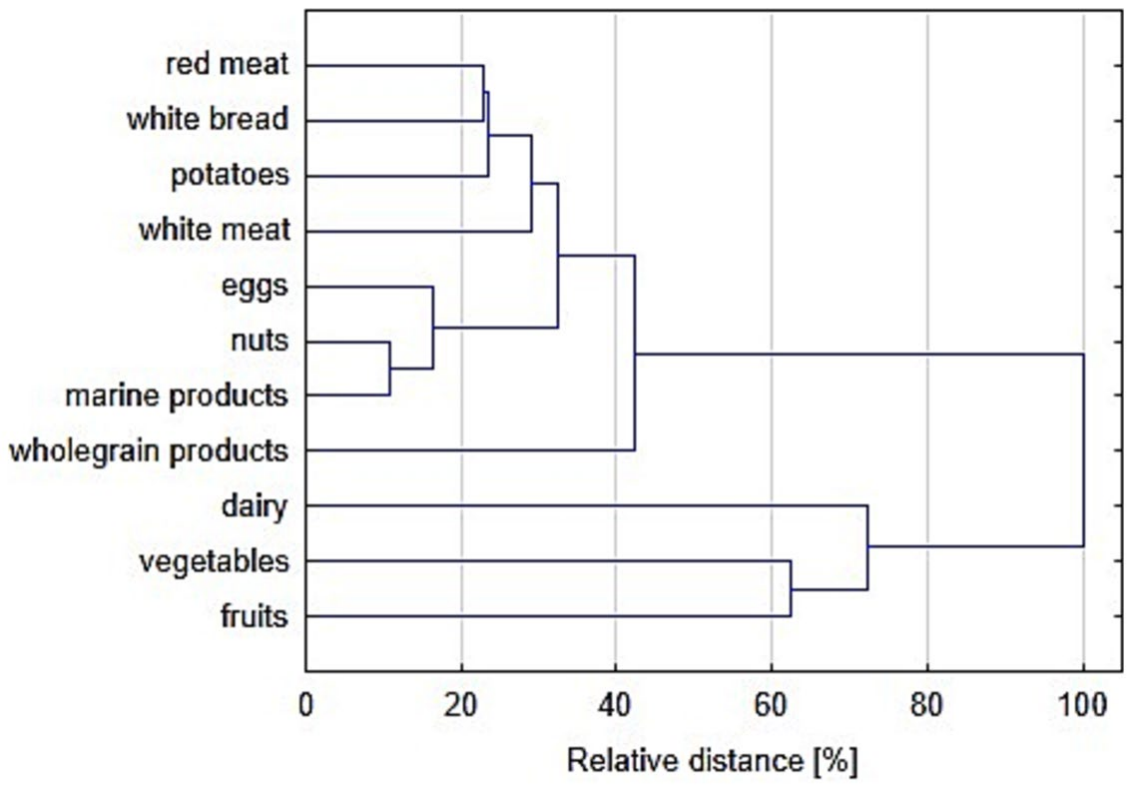
The results of the hierarchical agglomerative clustering between 11 consumed food categories. Euclidean distance and complete-linkage clustering methods were used.

Furthermore, with the use of the EFA, two unrelated patterns of food consumption were found as characterized by two orthogonal factors in the EFA. One of them could represent the food of overall high nutritional value and included wholegrain products, nuts, vegetables, fruits, as well as – to a lesser degree – marine products, white meat, dairy and eggs, while the other one could represent food of lower nutritional value and comprised potatoes, red meat and white bread. In the other words, people eating more vegetables, eat more wholegrain products, fruits and nuts; on the other hand people eating more red meat, eat more white bread and potatoes. The EFA results are presented in Figure 2 and are partially confirmed by hierarchical agglomerative clustering as reported in Figure 3.

Further analysis of the weighted sum of the products included in two abovementioned EFA-derived factors (factor loadings as weights in the equations) showed that products with higher nutritional value (Factor 1) were found to be significantly positively associated with higher conscientiousness, however, not with the other personality traits, in both raw and multivariate analysis. The results are presented in Table 4.

**Table 4:** Association between personality traits and health-related measures under stress.

	**Neuroticism**	**Extraversion**	**Openness**	**Agreeableness**	**Conscientiousness**
**Raw analysis**					
Factor 1^**a**^	−0.06 (−0.16 to 0.03)	0.05 (−0.04 to 0.14)	0.02 (−0.07 to 0.12)	0.02 (−0.08 to 0.12)	**0.16 (0.07 to 0.26)**
	pη^2^ = 0.4% *p* = 0.18	pη^2^ = 0.2% *p* = 0.30	pη^2^ = 0.0% *p* = 0.65	pη^2^ = 0.0% *p* = 0.68	**pη**^**2**^ **= 2.5% *p* = 0.0009**
Factor 2^**b**^	−0.08 (−0.18 to 0.02)	−0.05 (−0.15 to 0.04)	−0.01 (−0.11 to 0.08)	−0.06 (−0.16 to 0.03)	−0.10 (−0.20 to −0.00)
	pη^2^ = 0.6% *p* = 0.10	pη^2^ = 0.3% *p* = 0.26	pη^2^ = 0.0% *p* = 0.79	pη^2^ = 0.4% *p* = 0.20	pη^2^ = 1.0% *p* = 0.040
**Adjusted analysis**					
Factor 1^**a,c**^	−0.08 (−0.18 to 0.02)	0.04 (−0.05 to 0.14)	0.03 (−0.07 to 0.12)	0.01 (−0.09 to 0.11)	**0.16 (0.06 to 0.26)**
	pη^2^ = 0.5% *p* = 0.12	pη^2^ = 0.2% *p* = 0.37	pη^2^ = 0.1% *p* = 0.58	pη^2^ = 0.0% *p* = 0.84	**pη**^**2**^ **= 2.3% *p* = 0.0015**
Factor 2^**b,d**^	0.07 (−0.02 to 0.16)	−0.03 (−0.11 to 0.06)	−0.05 (−0.13 to 0.04)	−0.02 (−0.11 to 0.06)	−0.00 (−0.09 to 0.08)
	pη^2^ = 0.5% *p* = 0.12	pη^2^ = 0.1% *p* = 0.55	pη^2^ = 0.3% *p* = 0.27	pη^2^ = 0.1% *p* = 0.60	pη^2^ = 0.0% *p* = 0.96

Health- related behavior was expressed as factors obtained from the exploratory factor analysis (details in the text). The values in bold are considered statistically significant at the Benjamini and Hochberg corrected significance level of 0.0050 (false discovery rate 0.05).^a^EFA-derived representation of products with higher nutritional value.^b^EFA-derived representation of products with lower nutritional value.^c^beta coefficients (value of *p*s) for covariates: sex −0.05 (*p* = 0.28), socioeconomic status 0.00 (*p* = 0.92), number of inhabitants in the place of family residence 0.11 (*p* = 0.02) and any chronic disease −0.01 (*p* = 0.85).^d^beta coefficients (*value of p*s) for covariates: sex 0.52 (*p* < 0.0001), socioeconomic status −0.01 (*p* = 0.86), number of inhabitants in the place of family residence −0.07 (*p* = 0.10) and any chronic disease 0.03 (*p* = 0.49).

### 3.5. Sensitivity analysis in subgroups

In order to assess whether the reported effects are stable across different subgroups, we performed three sensitivity analyses. First one was done in a subgroup of people experiencing at least mild depressive or anxiety symptoms (PHQ-9 score ≥ 5 or GAD-7 score ≥ 5; *n* = 368, 82.9% of the whole sample) in the time preceding the stressful event, and the results showed that conscientiousness was still the only personality trait associated with healthier food consumption (Factor 1 in EFA) (adjusted beta 0.14, 95%CI 0.03 to 0.25, *p* = 0.012); also, there was no significant interaction between experiencing mental health symptoms (PHQ-9 score or GAD-7 score) and conscientiousness in predicting eating healthier foods (Factor 1 in EFA) (*p* = 0.78 and *p* = 0.70, respectively; covariate-adjusted analyses with all personality traits and the two-way interaction linearly included). The second sensitivity analysis included people having no endocrine or gastroenterological diseases (*n* = 375, 84.5%), which could have changed dietary patterns themselves; the effect in this regard was also similar to that of the main analysis (adjusted beta for conscientiousness 0.14, 95%CI 0.03 to 0.25, *p* = 0.010). The third sensitivity analysis was performed in the total sample, but with exclusion of chronic diseases as a covariate, due to the possibility that personality traits contribute to chronic diseases (e.g., development of psychiatric disorders), which could be better described as a mediator than a confounder. In this analysis the result turned out to be identical to the dependency found originally (adjusted beta for conscientiousness 0.16, 95%CI 0.06 to 0.26, *p* = 0.0015). Finally – as the validation of the FR tool suggested some inter-individual differences in its precision – the other sensitivity analysis was performed in a subgroup of students reporting high adherence to the FR (declaring no more than 10% of consumed food products to be omitted, *n* = 341, 76.8%). In this subgroup the association between consumption of healthy foods (Factor 1 in EFA) and conscientiousness was significantly positive (adjusted beta 0.16, 95%CI 0.05 to 0.27, *p* = 0.0062). All these results support versatility of the positive association between conscientiousness and healthy food consumption.

## 4. Discussion

The association between personality traits and health-related behavior has been a subject of many studies (12, 13, 31–35). Nevertheless, the recent systematic review on this topic indicated the need for further investigation involving particular populations (11). Due to overwhelming psychological stress experienced in everyday live (the COVID-19 pandemic, the war waged nearby), it was particularly interesting to assess this relationship in a severely stressed population. Our data indicated that higher conscientiousness may help in leading a generally healthier lifestyle in this population. Such association – although in line with many previous studies (34, 36–38) – is particularly interesting, as the studied population of medical students presented relatively high background level of conscientiousness; we found no ceiling effect in this regard. Even though conscientiousness was found to be positively associated with eating healthy food, there was no reverse dependency between conscientiousness and consumption of less healthy products.

Our findings are in line with the existing body of literature, despite the specific type of the population examined. The study performed among South Korean college students described a positive correlation between conscientiousness and extensive self-reported physical activity and lower BMI, which may be indicative of a healthier lifestyle (31). Another study carried out among patients with metabolic syndrome in Poland suggested an association between low conscientiousness and the tendency to develop metabolic syndrome which may be diet-induced (32). The role of conscientiousness in terms of health-related measures was also taken into consideration in a study on child obesity, where a positive correlation between higher conscientiousness and lower BMI was demonstrated. Additionally, the participants with a stronger expression of this personality trait had a tendency to engage more in physical activity and paid attention to their diet in terms of ingredients and regular meal rhythms (33). Furthermore, the study performed in a large sample of adult Estonians also found the link between higher conscientiousness and health-aware diet (13). A specific population of 70-year-olds also demonstrated higher conscientiousness correlated with a more favorable diet and lower BMI (35). From a different perspective, another study conducted among young adults showed an association between higher conscientiousness and greater plant-food consumption (12). It all means that the phenomenon of conscientiousness predisposing to healthier habits is very versatile and works under various circumstances.

Conscientiousness is defined as a personality trait that reflects tendencies to be hardworking, rule-following, determined and task-oriented, set on planning and achieving goals (39). It is also described as a foundation of self-discipline and the basis for regulation of internal urges, which may function as a source of control over health-related behaviors (32). This personality construct may even be a vital determinant of positive aging and general human capital (40). In this light, it becomes clear why conscientiousness, not other personality indices, predicts more favorable health behaviors. In general, medical students who were examined in this study are knowledgeable about healthy diet. However, their behavior does not necessarily reflect the knowledge. This phenomenon, referred to as the knowledge–behavior gap, may be less pronounced in highly conscientious people, particularly in an unusual context, such as examination session (41).

As high conscientiousness can lead to development of more favorable health behaviors, people with low conscientiousness may require special attention. First of all, it is important to note that people with lower conscientiousness are not devoid of this quality, it is simply weaker. Therefore, they are less likely to follow through with plans and are less motivated to achieve their goals (42). That is why it could be intriguing to explore whether personality may be modified. Although classically regarded as stable, personality traits can evolve and may be modified with specific interventions, as suggested by evidence presented in the literature (43). The possibility of personality modification is particularly the case in young adults, whose development is still in progress (44). Individuals with low conscientiousness should attempt to modify their lifestyle by introducing mechanisms that may not only improve conscientiousness attributes but also contribute to more effective stress management and a greater awareness when planning a diet. The most well-known and effective method that develops conscientiousness is the behavioral activation method (43). Applying this approach, an individual monitors his/her habits and behaviors on a daily basis and defines them in terms of importance – duty or pleasure. A person learns the structure of their day and is able to define the activities that they consider pleasurable or obligatory. Someone who is not good at planning or organization, through systematic work on learning the schedule of the day, observing at what times of the day and night they function better or perform activities more efficiently, will eventually be able to effectively manage not only their functioning, but also their diet.

When an individual does not plan to modify his or her own personality traits, e.g., conscientiousness, but wishes to improve diet, attention should be paid to issues such as self-regulation and adherence to nutritional standards (45). The ability to self-regulate is recognized by researchers as an element specific to the human race, one that distinguishes us from other species. Self-regulation is nothing other than the ability to change one’s own feelings, thoughts and actions. Thanks to it, individuals are capable of inhibiting, e.g., the process of gratification which can negatively affect our diet. By learning about the three stages of self-regulation, namely a sense of duty toward nutritional standards, observation of one’s own habits, and the ability to change one’s behavior when it is not in accordance with the standards, one can improve daily diet (45). Food standards are the norms, patterns, values or expectations that guide an individual in the process of self-regulation. The greater the commitment and loyalty to one’s own set objectives, the better an individual can expect to do when starting a healthy diet. An equally important aspect is self-efficacy (46) – the greater the belief in one’s own abilities, the easier it is to achieve a goal. It is important to note, however, that the nutritional patterns we follow are also socially and culturally conditioned (45). It is worth paying attention to the characteristics of stimuli associated with general diet that reach us. We should analyze how we re-late to them, and how strongly they construct our lifestyle.

Apart from conscientiousness, the literature reports the connection between neuroticism and the tendency to consume unhealthy diet (47–50). It is manifested by a preference for sugar-containing products (sweets, chocolate, and soft drinks) (47–49). A few of the studies also found the association between higher neuroticism and increased salty food intake, along with reduced fish and vegetables consumption (47, 50). Our results did not confirm the link between neuroticism and unhealthy habits, however, this might have been a false-negative effect as some *p*-values for this link were relatively low. Especially the association between neuroticism and BMI presents some but inconclusive trend. This uncertain outcome might be a result of the fact that BMI of great majority of the participants falls within the normal range, hence the low variance and lack of discrimination of this feature by personality traits was observed. It is also worth noting that considering all the stressors reflected in high degree of anxiety and depression symptoms, the studied population appeared to collectively present “neurotic behavior,” which might stifle potential dependencies involving this personality trait.

As for sociodemographic factors, among the surveyed students, there was an association found between sex and the products consumed. Men more often included such products as red and white meat, eggs, light bread and potatoes in their diet. Women, on the other hand, consumed more vegetables, fruits and nuts. These results are consistent with those obtained in other studies (51, 52) suggesting that men consume more animal-derived and fatty meals and are less likely to become vegetarians, whereas women present a greater engagement in healthy nutrition. This difference may also simply result from higher energy demand in males (30). Additionally, our results showed a positive correlation between the size of the place of residence of the family of origin and the products consumed or the lifestyle. Students coming from larger cities consumed more vegetables, fish and seafood, and paid more attention to physical activity. This may be intriguing and counterintuitive, however, it finds some confirmation in the other study linking the residence in rural areas with greater consumption of fats and oils than that observed in bigger cities (53).

The present study has several limitations. Firstly, to a large degree it is a secondary analysis of an existing database – a participant subsample has been already described with regard to consumption of fermented food and mental health in previous papers (14, 15). As such, the certainty of the reported findings may be diminished and the choice of specific food groups included in the questionnaire was dictated by the requirements of that primary study. Moreover, the sample size is relatively small for detecting weaker associations between personality traits and behavior. Secondly, personality traits were assessed based on a shortened version of a questionnaire (Big Five Inventory-Short), which relies on three items for each personality trait only. Nevertheless, the questionnaire assures its validity (18, 19). The internal consistency measures were deemed acceptable for most personality traits (54) with the exemption of Agreeableness for which Cronbach’s alpha was impaired; in consequence the results for Agreeableness should be regarded with caution. Thirdly, our Food Record questionnaire neither includes all the food groups (e.g., data on consumption of sweets, coffee, alcohol and drugs was not collected, which might affect the consumption of products with low nutritional value), nor evaluates the ways in which the meals were prepared; however, the Food Record measure was additionally validated to assure its high accuracy. Moreover, we did not examine students’ inhabiting conditions – whether they live with their parents, with roommates or on their own, which might have an impact on their daily dietary choices. Finally, the study is based on self-reported data (in particular physical activity might have been collected through accelerometers), and therefore may be more declarative and socially desirable.

## 5. Conclusion

Severely stressed medical students showing high conscientiousness tend to present healthier behaviors expressed as dietary patterns (more consumption of vegetables, wholegrain products, fruits and nuts) and avoiding cigarette smoking. The results are consistent with literature reports on other populations, which may suggest high versatility of the phenomenon, even in the population of high background conscientiousness. Interventions aimed at improving lifestyle habits in students with lower conscientiousness might be useful.

## Data availability statement

The dataset for this article is not publicly available due to concerns regarding participant anonymity. Requests to access the dataset should be directed to the corresponding author.

## Ethics statement

The studies involving humans were approved by Bioethics Committee of the Medical University of Lodz, Poland. The studies were conducted in accordance with the local legislation and institutional requirements. The participants provided their written informed consent to participate in this study.

## Author contributions

JT: Conceptualization, Data curation, Formal analysis, Validation, Visualization, Writing – original draft, Writing – review & editing. AK: Writing – original draft. JK: Writing – original draft. EK: Funding acquisition, Validation, Writing – review & editing. MK: Conceptualization, Data curation, Formal analysis, Funding acquisition, Investigation, Methodology, Project administration, Resources, Supervision, Validation, Visualization, Writing – review & editing.
