# Supplementary material for: Personality traits and health-related behaviors in medical students facing a stressful event
Source: Front Public Health. 2023 Oct 10;11:1256883. doi: 10.3389/fpubh.2023.1256883 (PMC10602886; doi:10.3389/fpubh.2023.1256883)
Supplement: Supplementary file 1 [file Table_1.DOCX]

Supplementary Material

# Food Record (FR) validation.

## Materials and Methods

Criterion validity of the FR tool was assessed to estimate the accuracy and precision of the applied measure in predicting the real mass of consumed food products. Pairs of medical students not involved in the study were invited. One of the person in a pair was asked to weigh several food products with electronic kitchen scale for the other person, whose task was to estimate the mass of food portion with the use of the FR tool. Then, mean error (ME) was calculated to investigate any systematic error and estimate accuracy, whereas mean relative absolute error (MRAE) was used to assess precision of the FR tool.

## Results

Five pairs of people took part in criterion validation of the FR tool. The mean age of those who estimated the food portions with the FR was 23.4 ± 0.9 years and two of them were male. They assessed the masses of 165 food portions in total (range 12 to 55 for a person). ME of the estimations was -0.0 (95% CI -2.9 to 2.9) grams, suggesting no systematic error made with the FR and satisfactory accuracy (p=0.99, one-sample t-test). There was also no evidence for inter-individual difference in ME (F(4,160)=1.49, p=0.21), suggesting good accuracy between the tested people. Some imprecision of the FR measure was detected as MRAE was 16.7% (95%CI 13.5% to 20.0%), indicating the use of FR yields no more than a fifth of absolute deviations from real food portion masses. Also, substantial inter-individual variations were detected in the precision as there was a difference in MRAE between the tested people (F(4,160)=4.22, p=0.0028), suggesting some people used FR tool more and some of them less precisely.

# Supplementary Table - Consumption of all food products included in the Food Record questionnaire in the seven-day pre-exam period.

| **FR item number** | **Food product** | **Seven-day consumption [gram]** | |
| --- | --- | --- | --- |
|  |  | **Median (1^st-^3^rd^ quartiles)** | **Mean (standard deviation)** |
| 1a | Red meat | 380.8 (134.4-683.2) | 474.2 (445.1) |
| 1b | White meat | 336.0 (75.6-697.2) | 460.5 (464.4) |
| 1c | Fatty fish | 0.0 (0.0-112.0) | 8.9 (156.7) |
| 1d | Other fish | 0.0 (0.0-36.8) | 44.8 (95.8) |
| 1e | Fish oil | 0.0 (0.0-0.0) | 5.9 (15.6) |
| 1f | Seafood | 0.0 (0.0-0.0) | 8.9 (41.5) |
| 2a | Milk | 541.0 (154.2-1047.2) | 766.2 (816.3) |
| 2b | Cottage cheese | 95.2 (0.0-235.0) | 177.0 (249.2) |
| 2c | Cheese | 134.4 (50.4-235.2) | 168.3 (151.7) |
| 2d | Yogurt, kefir, soured milk | 280.0 (28.0-616.0) | 434.1 (524.6) |
| 2e | Eggs | 246.4 (123.2-409.4) | 288.3 (238.0) |
| 3a | Light bread | 514.3 (230.7-842.9) | 569.2 (425.4) |
| 3b | Wholemeal bread, graham | 151.2 (0.0-392.0) | 249.8 (286.6) |
| 3c | Cereal, groats, whole grain noodle | 280.0 (66.7-560.0) | 398.2 (462.1) |
| 3d | Muesli | 0.0 (0.0-82.9) | 70.1 (135.3) |
| 3e | White rice | 47.6 (0.0-185.7) | 139.8 (234.5) |
| 3f | Unpasteurized kvass and beer | 0.0 (0.0-0.0) | 43.9 (197.1) |
| 3g | Wholemeal flour | 0.0 (0.0-112.0) | 82.1 (146.8) |
| 4a | Potatoes | 426.9 (201.6-672.0) | 469.7 (369.0) |
| 4b | Carrot, parsley, celery | 52.5 (0.0-134.0) | 108.5 (153.7) |
| 4c | Beetroots | 0.0 (0.0-56.0) | 60.3 (166.7) |
| 4d | Raw cucumber | 62.2 (0.0-156.8) | 114.4 (156.8) |
| 4d | Pickled cucumber and pickling juice | 0.0 (0.0-82.0) | 72.0 (151.5) |
| 4f | Cabbage | 0.0 (0.0-168.0) | 122.5 (259.2) |
| 4g | Sauerkraut and pickling juice | 0.0 (0.0-0.0) | 31.0 (93.7) |
| 4h | Other fermented vegetables and their pickling juice | 0.0 (0.0-0.0) | 6.5 (53.2) |
| 4i | Onion, leek, garlic | 67.2 (11.2-173.6) | 126.5 (163.9) |
| 4j | Leguminous vegetables | 0.0 (0.0-134.4) | 109.9 (199.1) |
| 4k | All other vegetables | 940.8 (537.1-1512.0) | 1096.6 (739.2) |
| 5a | Apples | 67.2 (0.0-408.8) | 297.3 (537.9) |
| 5b | Citrus | 0.0 (0.0-133.9) | 164.2 (411.0) |
| 5c | Bananas | 164.0 (0.0-403.2) | 267.0 (309.7) |
| 5d | All other fruits | 762.8 (243.6-1271.2) | 865.5 (743.6) |
| 5e | Nuts | 15.7 (0.0-67.2) | 50.9 (81.6) |

# Supplementary Table - Association between personality traits and health-related behavior under stress – raw analysis.

# Consumption of selected food products, physical activity, general quality of diet and BMI were assessed with the use of multivariate linear regression and standardized ß with 95% confidence intervals (CI) and partial η-square were presented as effect size measures. Cigarettes use was assessed with the use of multivariate logistic regression and odds ratio with 95% CI were presented as effect size measures. Each row in the table depicts the results of a single multivariate model.

| **Health-related  behavior** | **Effect size measure, *p*-value** | | | | |
| --- | --- | --- | --- | --- | --- |
|  | **Neuroticism** | **Extraversion** | **Openness** | **Agreeableness** | **Conscientious-**  **ness** |
| **Consumption of selected food products – primary outcome** | | | | | |
| **Red meat** | -0.15 (-0.24 to -0.05) | -0.02 (-0.12 to 0.08) | 0.02 (-0.08 to 0.12) | -0.04 (-0.14 to 0.06) | -0.08 (-0.18 to 0.02) |
|  | pη^2^=2.1% *p*=0.0024 | pη^2^=0.0% *p*=0.69 | pη^2^=0.0% *p*=0.72 | pη^2^=0.0% *p*=0.43 | pη^2^=0.0% *p*=0.10 |
| **White meat** | 0.044 (-0.05 to 0.14) | -0.01 (-0.10 to 0.09) | -0.07 (-0.17 to 0.03) | -0.02 (-0.12 to 0.08) | 0.03 (-0.07 to 0.13) |
|  | pη^2^=0.2% *p*=0.37 | pη^2^=0.0% *p*=0.92 | pη^2^=0.5% *p*=0.15 | pη^2^=0.0% *p*=0.71 | pη^2^=0.1% *p*=0.53 |
| **Marine products** | -0.10 (-0.19 to 0.00) | 0.13 (0.04 to 0.23) | 0.06 (-0.03 to 0.16) | -0.08 (-0.17 to 0.02) | 0.10 (-0.01 to 0.19) |
|  | pη^2^=0.9% *p*=0.050 | pη^2^=1.7% *p*=0.007 | pη^2^=0.4% *p*=0.19 | pη^2^=0.6% *p*=0.12 | pη^2^=0.8% *p*=0.067 |
| **Dairy** | -0.04 (-0.14 to 0.06) | 0.01 (-0.09 to 0.11) | 0.03 (-0.07 to 0.12) | -0.04 (-0.14 to 0.06) | 0.10 (-0.00 to 0.19) |
|  | pη^2^=0.2% *p*=0.42 | pη^2^=0.0% *p*=0.85 | pη^2^=0.1% *p*=0.61 | pη^2^=0.1% *p*=0.43 | pη^2^=0.8% *p*=0.062 |
| **Eggs** | -0.01 (-0.10 to 0.09) | -0.05 (-0.14 to 0.05) | -0.01 (-0.10 to 0.09) | 0.03 (-0.07 to 0.13) | 0.06 (-0.04 to 0.16) |
|  | pη^2^=0.0% *p*=0.89 | pη^2^=0.2% *p*=0.33 | pη^2^=0.0% *p*=0.91 | pη^2^=0.1% *p*=0.51 | pη^2^=0.3% *p*=0.24 |
| **Wholegrain products** | -0.03 (-0.12 to 0.07) | 0.02 (-0.08 to 0.11) | 0.035 (-0.06 to 0.13) | -0.02 (-0.12 to 0.07) | 0.14 (0.05 to 0.24) |
|  | pη^2^=0.5% *p*=0.57 | pη^2^=0.1% *p*=0.74 | pη^2^=0.1% *p*=0.49 | pη^2^=0.1% *p*=0.62 | pη^2^=1.9% *p*=0.0036 |
| **Light bread** | -0.10 (-0.19 to -0.00) | -0.08 (-0.18 to 0.01) | -0.03 (-0.12 to 0.07) | -0.04 (-0.14 to 0.06) | -0.14 (-0.23 to -0.04) |
|  | pη^2^=0.9% *p*=0.046 | pη^2^=0.7% *p*=0.087 | pη^2^=0.1% *p*=0.60 | pη^2^=0.2% *p*=0.42 | pη^2^=1.7% *p*=0.0055 |
| **Potatoes** | 0.02 (-0.08 to 0.12) | -0.03 (-0.12 to 0.07) | 0.04 (-0.06 to 0.14) | -0.06 (-0.15 to 0.04) | -0.01 (-0.10 to 0.09) |
|  | pη^2^=0.0% *p*=0.71 | pη^2^=0.1% *p*=0.57 | pη^2^=0.2% *p*=0.39 | pη^2^=0.3% *p*=0.27 | pη^2^=0.0% *p*=0.90 |
| **Vegetables** | -0.05 (-0.15 to 0.04) | 0.05 (-0.05 to 0.14) | 0.06 (-0.04 to 0.16) | 0.07 (-0.02 to 0.17) | 0.07 (-0.02 to 0.17) |
|  | pη^2^=0.3% *p*=0.28 | pη^2^=0.2% *p*=0.33 | pη^2^=0.4% *p*=0.21 | pη^2^=0.5% *p*=0.13 | pη^2^=0.5% *p*=0.13 |
| **Fruits** | -0.07 (-0.17 to 0.02) | 0.02 (-0.07 to 0.12) | -0.07 (-0.16 to 0.03) | -0.01 (-0.10 to 0.09) | 0.10 (0.00 to 0.20) |
|  | pη^2^=0.5% *p*=0.15 | pη^2^=0.0% *p*=0.65 | pη^2^=0.4% *p*=0.19 | pη^2^=0.0% *p*=0.89 | pη^2^=0.9% *p*=0.042 |
| **Nuts** | -0.03 (-0.13 to 0.06) | -0.02 (-0.11 to 0.08) | 0.01 (-0.08 to 0.11) | 0.00 (-0.10 to 0.10) | 0.04 (-0.06 to 0.14) |
|  | pη^2^=0.3% *p*=0.48 | pη^2^=0.3% *p*=0.75 | pη^2^=0.3% *p*=0.77 | pη^2^=0.3% *p*=0.96 | pη^2^=0.3% *p*=0.41 |
| **Other health-related measures – secondary outcomes** | | | | | |
| **Pre-exam physical activity ^a^** | -0.11 (-0.21 to -0.02) | -0.01 (-0.11 to 0.08) | 0.09 (-0.01 to 0.18) | -0.00 (-0.10 to 0.10) | 0.03 (-0.06 to 0.13) |
|  | pη^2^=1.1% *p*=0.023 | pη^2^=0.0% *p*=0.78 | pη^2^=0.7% *p*=0.087 | pη^2^=0.0% *p*=0.92 | pη^2^=0.1% *p*=0.50 |
| **Pre-exam general quality of diet ^b^** | 0.02 (-0.08 to 0.11) | 0.07 (-0.02 to 0.17) | 0.01 (-0.09 to 0.10) | -0.03 (-0.13 to 0.06) | **0.20 (0.11 to 0.30)** |
|  | pη^2^=0.0% *p*=0.69 | pη^2^=0.5% *p*=0.14 | pη^2^=0.0% *p*=0.89 | pη^2^=0.1% *p*=0.48 | **pη^2^=3.8% *p<*0.0001** |
| **Cigarettes ^c^** | 0.96 (0.87 to 1.06) | 1.05 (0.95 to 1.16) | 0.94 (0.85 to 1.05) | 0.89 (0.79 to 1.00) | **0.82 (0.74 to 0.91)** |
|  | *p*=0.44 | *p*=0.30 | *p*=0.33 | *p*=0.047 | ***p*=0.00033** |
| **BMI** | **-0.21 (-0.30 to -0.11)** | -0.05 (-0.15 to 0.04) | -0.01 (-0.11 to 0.08) | -0.02 (-0.12 to 0.07) | -0.14 (-0.24 to -0.05) |
|  | **pη^2^=4.1% *p<*0.0001** | pη^2^=0.3% *p*=0.28 | pη^2^=0.0% *p*=0.78 | pη^2^=0.1% *p*=0.63 | pη^2^=1.9% *p*=0.0034 |

BMI – body-mass index

The values in bold are considered statistically significant at the Benjamini and Hochberg corrected significance level of 0.0014 (false discovery rate 0.05).

a - Physical activity was expressed in the five-point semantic differential scale from 1 (“I have no physical activity at all”) to 5 (“I play sport intensively 5 times a week”), with the midpoint of 3.

b - General quality of diet was expressed as the number of points in the reverse-coded Starting the Conversation scale in the range from 0 (maximally unhealthy diet) to 16 (maximally healthy diet), with the midpoint of 8; Cronbach’s alpha assessed in the study sample was 0.55.

c - Fraction of the participants reporting either traditional cigarette smoking or e-cigarette use.

# Supplementary Table - Association between the analyzed covariates and health-related behavior in the multivariate models.

# Consumption of selected food products, physical activity, general quality of diet and BMI were assessed with the use of multivariate linear regression and standardized ß with 95% confidence intervals (CI) and partial η-square were presented as effect size measures. Cigarettes use was assessed with the use of multivariate logistic regression and odds ratio with 95% CI were presented as effect size measures. Each row in the table depicts the results of a single multivariate model.

|  | **Covariates** | | | |
| --- | --- | --- | --- | --- |
|  | **Sex  (k=0, m=1)** | **Socioeconomic status** | **Number of inhabitants in the place of family residence** | **Any chronic disease** |
| **Red meat** | **0.51 (0.42 to 0.59)** | -0.00 (-0.09 to 0.08) | -0.07 (-0.15 to 0.01) | -0.01 (-0.09 to 0.07) |
|  | **pη2=23.3%** ***p*<0.0001** | pη2=0.0% *p*=0.95 | pη2=0.6% *p*=0.095 | pη2=0.0% *p*=0.82 |
| **White meat** | **0.27 (0.17 to 0.36)** | 0.09 (-0.00 to 0.18) | -0.01 (-0.10 to 0.09) | 0.03 (-0.07 to 0.12) |
|  | **pη2=6.6%** ***p*<0.0001** | pη2=0.8% *p*=0.059 | pη2=0.0% *p*=0.91) | pη2=0.1% *p*=0.60 |
| **Marine products** | 0.08 (-0.02 to 0.17) | 0.06 (-0.03 to 0.16) | 0.09 (0.00 to 0.19) | -0.03 (-0.13 to 0.06) |
|  | pη2=0.5% *p*=0.13 | pη2=0.4% *p*=0.19 | pη2=0.9% *p*=0.047 | pη2=0.1% *p*=0.50 |
| **Dairy** | 0.12 (0.02 to 0.22) | -0.05 (-0.14 to 0.05) | -0.00 (-0.10 to 0.09) | -0.00 (-0.10 to 0.09) |
|  | pη2=1.3% *p*=0.018 | pη2=0.2% *p*=0.32 | pη2=0.0% *p*=0.99 | pη2=0.0% *p*=0.97 |
| **Eggs** | **0.22 (0.12 to 0.31)** | 0.04 (-0.05 to 0.13) | 0.07 (-0.03 to 0.16) | -0.05 (-0.14 to 0.05) |
|  | **pη2=4.1% *p*<0.0001** | pη2=0.2% *p*=0.41 | pη2=0.4% *p*=0.16 | pη2=0.2% *p*=0.35 |
| **Wholegrain products** | 0.03 (-0.07 to 0.13) | -0.02 (-0.11 to 0.08) | 0.00 (-0.09 to 0.10) | 0.00 (-0.09 to 0.10) |
|  | pη2=0.1% *p*=0.51 | pη2=0.0% *p*=0.70 | pη2=0.0% *p*=0.96 | pη2=0.0% *p*=0.94 |
| **Light bread** | **0.32 (0.22 to 0.41)** | -0.07 (-0.16 to 0.02) | 0.01 (-0.08 to 0.10) | 0.00 (-0.09 to 0.09) |
|  | **pη2=9.3% *p*<0.0001** | pη2=0.5% *p*=0.14 | pη2=0.0% *p*=0.90 | pη2=0.0% *p*=0.93 |
| **Potatoes** | **0.23 (0.13 to 0.33)** | -0.01 (-0.10 to 0.08) | -0.08 (-0.18 to 0.01) | 0.09 (-0.00 to 0.19) |
|  | **pη2=4.8%** ***p*<0.0001** | pη2=0.0% *p*=0.86 | pη2=0.7% *p*=0.074 | pη2=0.8% *p*=0.055 |
| **Vegetables** | -0.12 (-0.22 to -0.03) | 0.02 (-0.07 to 0.11) | **0.16 (0.07 to 0.25)** | -0.00 (-0.10 to 0.09) |
|  | pη2=1.4% *p*=0.013 | pη2=0.0% *p*=0.65 | **pη2=2.6% *p*=0.0007** | pη2=0.0% *p*=0.97 |
| **Fruits** | -0.10 (-0.20 to -0.00) | -0.03 (-0.13 to 0.06) | 0.04 (-0.05 to 0.14) | -0.02 (-0.12 to 0.07) |
|  | pη2=1.0% *p*=0.041 | pη2=0.1% *p*=0.50 | pη2=0.2% *p*=0.35 | pη2=0.0% *p*=0.65 |
| **Nuts** | -0.11 (-0.21 to -0.01) | 0.10 (0.00 to 0.19) | -0.02 (-0.11 to 0.08) | 0.02 (-0.08 to 0.11) |
|  | pη2=1.1% *p*=0.027 | pη2=0.9% *p*=0.044 | pη2=0.0% *p*=0.73 | pη2=0.0% *p*=0.71 |
| **Physical activity ^a^** | 0.08 (-0.02 to 0.17) | -0.07 (-0.16 to 0.03) | 0.10 (0.01 to 0.19) | 0.07 (-0.03 to 0.16) |
|  | pη2=0.5% *p*=0.13 | pη2=0.5% *p*=0.15 | pη2=1.0% *p*=0.038 | pη2=0.4% *p*=0.18 |
| **General quality of diet ^b^** | -0.07 (-0.03 to 0.16) | 0.01 (-0.10 to 0.08) | 0.01 (-0.11 to 0.08) | -0.08 (-0.02 to 0.17) |
|  | pη2=0.4% *p*=0.19 | pη2=0.0% *p*=0.83 | pη2=0.0% *p*=0.79 | pη2=0.6% *p*=0.11 |
| **Cigarettes ^c^** | 2.35 (1.00 to 5.55) | 1.22 (0.52 to 2.88) | 0.83 (0.56 to 1.22) | 0.91 (0.38 to 2.16) |
|  | *p*=0.050 | *p*=0.63 | *p*=0.33 | *p*=0.83 |
| **BMI** | **0.34 (0.25 to 0.43)** | 0.06 (-0.03 to 0.15) | -0.03 (-0.11 to 0.06) | 0.05 (-0.04 to 0.14) |
|  | **pη2=10.8% *p*<0.0001** | pη2=0.4% *p*=0.20 | pη2=0.1% *p*=0.55 | pη2=0.3% *p*=0.24 |

BMI – body-mass index

The values in bold are considered statistically significant at the Benjamini and Hochberg corrected significance level of 0.0037 (false discovery rate 0.05).

a - Physical activity was assessed in the pre-exam stage of the study and is expressed in the five-point semantic differential scale from 1 (“I have no physical activity at all”) to 5 (“I play sport intensively 5 times a week”), with the midpoint of 3.

b - General quality of diet was assessed in the pre-exam stage of the study and is expressed as the number of points in the reverse-coded Starting the Conversation scale in the range from 0 (maximally unhealthy diet) to 16 (maximally healthy diet), with the midpoint of 8; Cronbach’s alpha assessed in the study sample was 0.55.

c – Odds ratio with 95% confidence intervals for reporting either traditional cigarette smoking or e-cigarette use.
